# Developmental Diet Alters the Fecundity–Longevity Relationship and Age-Related Gene Expression in *Drosophila melanogaster*

**DOI:** 10.1093/gerona/glad199

**Published:** 2023-08-16

**Authors:** David H Collins, David C Prince, Jenny L Donelan, Tracey Chapman, Andrew F G Bourke

**Affiliations:** School of Biological Sciences, University of East Anglia, Norwich, UK; School of Biological Sciences, University of East Anglia, Norwich, UK; School of Biological Sciences, University of East Anglia, Norwich, UK; School of Biological Sciences, University of East Anglia, Norwich, UK; School of Biological Sciences, University of East Anglia, Norwich, UK; (Biological Sciences Section)

**Keywords:** Aging, Eusociality, Life history, mRNA-seq, Nutrition

## Abstract

The standard evolutionary theory of aging predicts a negative relationship (trade-off) between fecundity and longevity. However, in principle, the fecundity–longevity relationship can become positive in populations in which individuals have unequal resources. Positive fecundity–longevity relationships also occur in queens of eusocial insects such as ants and bees. Developmental diet is likely to be central to determining trade-offs as it affects key fitness traits, but its exact role remains uncertain. For example, in *Drosophila melanogaster*, changes in adult diet can affect fecundity, longevity, and gene expression throughout life, but it is unknown how changes in developmental (larval) diet affect fecundity–longevity relationships and gene expression in adults. Using *D. melanogaster*, we tested the hypothesis that varying developmental diets alters the directionality of fecundity–longevity relationships in adults, and characterized associated gene expression changes. We reared larvae on low (20%), medium (100%), and high (120%) yeast diets, and transferred adult females to a common diet. We measured fecundity and longevity of individual adult females and profiled gene expression changes with age. Adult females raised on different larval diets exhibited fecundity–longevity relationships that varied from significantly positive to significantly negative, despite minimal differences in mean lifetime fertility or longevity. Treatments also differed in age-related gene expression, including for aging-related genes. Hence, the sign of fecundity–longevity relationships in adult insects can be altered and even reversed by changes in larval diet quality. By extension, larval diet differences may represent a key mechanistic factor underpinning positive fecundity–longevity relationships observed in species such as eusocial insects.

The standard evolutionary theory of aging predicts that, as individuals grow older, selection for increased survivorship declines with age (1). Therefore, individuals experience the age-related decrease in performance and survivorship that defines aging (senescence) (2). Additionally, given finite resources, individuals should optimize relative investment between reproduction and somatic maintenance (3). This causes trade-offs between reproduction and longevity (4,5) with elevated reproduction often incurring costs to longevity (the costs of reproduction) (6). Such trade-offs and costs are evident in the negative fecundity–longevity relationships observed in many species.

Although a negative fecundity–longevity relationship is typical, fecundity and longevity can become uncoupled (7) and some species or populations may exhibit positive fecundity–longevity relationships (4). This can occur for several reasons. First, in *Drosophila melanogaster*, mutations can increase longevity without apparent reproductive costs (8–11), particularly mutations in the conserved insulin/insulin-like growth factor signaling and target of rapamycin network (IIS-TOR). This network regulates nutrient sensitivity and is an important component of aging across diverse taxa (2,12).

Second, fecundity and longevity can become uncoupled when there is asymmetric resourcing between individuals (13,14). Within a population, well-resourced individuals may have higher fecundity and longevity than poorly resourced individuals, reversing the usual negative fecundity–longevity relationship. However, because costs of reproduction are not abolished even in well-resourced individuals (13,14), a within-individual trade-off between fecundity and longevity remains present.

Third, fecundity and longevity can become uncoupled within and between the castes of eusocial insects (15–18), that is, species such as ants, bees, wasps, and termites with a long-lived reproductive caste (queens or kings) and a short-lived non- or less reproductive caste (workers) (19–21). In some species, queens appear to have escaped costs of reproduction completely (22–25). This may have been achieved through rewiring the IIS-TOR network (12,26), which forms part of the TOR/IIS-juvenile hormone-lifespan and fecundity (TI-J-LiFe) network hypothesized to underpin aging and longevity in eusocial insects by Korb et al. (27).

*Drosophila melanogaster* is an important model species for exploring the effect of diet on fecundity and longevity. In this species, dietary restriction extends adult life span if either adult diets (28–30) or larval diets (31–34) are restricted. Differences in diet quality can also decouple fecundity–longevity relationships in adults (30,35). For example, longevity is maximized on a low protein:carbohydrate diet and reproduction is maximized on a high protein:carbohydrate diet (30,35). Changes in adult diets are accompanied by age-related transcriptional changes (35–37) and dietary effects might be underpinned by downregulation of IIS-TOR signaling (38). *Drosophila melanogaster* larvae reared on restricted diets exhibit longer development times, lower development success, and smaller body sizes, but effects on fecundity and/or longevity can be positive or negative (32,33,39,40). Additionally, larval and adult diets may interact to have contrasting effects on adult life-history traits (32,39). As larval diet also determines key fitness traits (eg, body size), these results suggest that variation in the larval diet could be important in shaping the relationship between adult fecundity and longevity (39).

To date, no study has explored whether *D. melanogaster* experiencing different larval diets differ in the directionality of the fecundity–longevity relationship as adults. In particular, it is not known whether, when larval diet is manipulated, the negative fecundity–longevity relationship usually found in *D. melanogaster* females (4) can be reversed so that it becomes positive. Establishing this is critical to understanding the mechanisms underpinning fecundity–longevity relationships. In addition, larval diet could be a key untested determinant of the directionality of the fecundity–longevity relationship in species such as eusocial insects. Under this hypothesis, which is based on the concept of asymmetric resourcing, abundant larval resources may amplify genetic, environmental, or stochastic differences in individual quality. This could cause high-quality individuals (able to capitalize on increased larval resources) to exhibit high adult fecundity and longevity and low-quality individuals (unable to capitalize on increased larval resources) to exhibit low fecundity and longevity, causing a positive fecundity–longevity relationship (18). These patterns are observed in eusocial insect queens, which receive high-quality larval diets compared to workers and exhibit a positive fecundity–longevity relationship (18,41). Therefore, the hypothesis predicts that, other things equal, a superior larval diet generates a positive fecundity–longevity relationship.

Accordingly, our first aim was to determine the effect of larval diet on the directionality of the fecundity–longevity relationship in adult *D. melanogaster* females. We reared *D. melanogaster* larvae on 3 treatment diets: low-quality (20% SYA, ie, Sugar Yeast Agar), medium-quality (100% SYA), and high-quality (120% SYA) diets (henceforth, L, M, and H treatments, respectively). We then reared the adult female flies eclosing from all 3 treatment diets on a different, standardized, common garden adult diet (110% SYA) and measured the effect of treatment (ie, larval diet) on individual adult longevity (number of days lived posteclosion) and fertility (ie, realized fecundity, as measured by egg production and offspring production). As larval diet affects development time, development success, and adult body size, we also measured these traits. We used females because this permitted direct measures of fertility and because females are more responsive to diet in their life-history traits than males (39). We hypothesized that the differences in larval diet would change the directionality of the fecundity–longevity relationship within each treatment. More specifically, we hypothesized that the fecundity–longevity relationship would be either positive, or less negative, in the H females (reared on a high-quality larval diet) compared to the M and L females (reared on medium- and low-quality larval diets, respectively).

Our second aim was to determine whether changing the directionality of the fecundity–longevity relationship would affect the expression of age-related genes (ie, genes that change expression with time). *Drosophila melanogaster* females show age-related transcriptional changes covarying with reproductive senescence (36,37) and adult diet (35). However, the genetic correlates of changes in the directionality of the fecundity–longevity relationship following manipulation of larval diet have not previously been investigated. We used mRNA-seq to sequence transcriptomes from head, fat body (abdomen minus gut and ovaries), and ovaries of females from the M and H treatments (as these 2 treatments showed the most pronounced difference in the directionality of the fecundity–longevity relationship) at 2 relative time intervals, that is, after 10% and 60% of females in each treatment had died (representing low and high mortality, respectively). We hypothesized that a change in the directionality of the fecundity–longevity relationship would alter the pattern of gene-expression change with relative age in each of these treatments. In comparisons with genes in the GenAge database (42), which contains experimentally validated *D. melanogaster* genes associated with aging, and in the TI-J-LiFe network (27), we also tested whether the expression of aging-related genes (ie, genes in pathways that directly affect aging) covaried with the directionality of the fecundity–longevity relationship.

## Method

### Fly Rearing and Sample Collection

**Figure 1.**
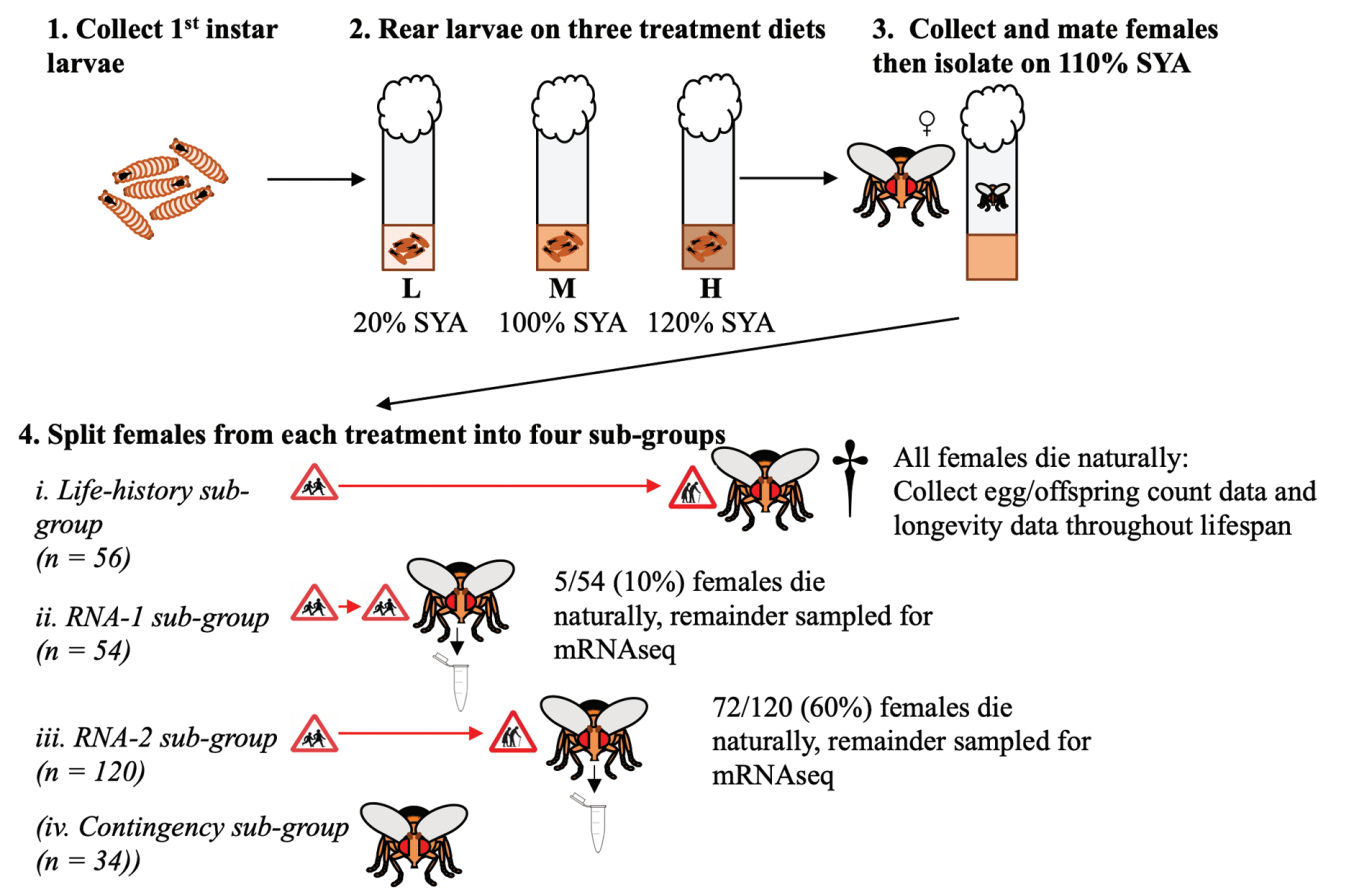
Outline of experimental design to determine the effect of larval diet (treatment) on the fecundity–longevity relationship and age-related gene expression of adult female *Drosophila melanogaster*. Flies were reared as larvae (of both sexes) on 3 treatment diets: L: low quality, 20% Sugar Yeast Agar (SYA); M: medium quality, 100% SYA; H: high quality, 120% SYA. Adult males were discarded or used for mating and adult females, after mating, were maintained on a common 110% SYA diet. Females were split into 4 subgroups per treatment: life-history subgroup: used to provide fertility and longevity data; RNA-1 subgroup: used to provide gene expression data following 10% adult mortality; RNA-2 subgroup used to provide gene expression data following 60% adult mortality; contingency subgroup: used to replace females that died during initial set-up and RNA females accidentally lost during handling. Horizontal arrows in section 4 represent the relative longevity of each subgroup before the last individual died or was sampled. Final sample sizes were: L: life-history (*n* = 56), RNA-1 (*n* = 54), RNA-2 (*n* = 120); M: life-history (*n* = 45), RNA-1 (*n* = 50), RNA-2 (*n* = 92); H: life-history (*n* = 56), RNA-1 (*n* = 54), RNA-2 (*n* = 120). See Method section for full details.

We reared females on different diets (20% (L), 100% (M), and 120% (H) standard SYA) as larvae, transferred them to a common diet (110% SYA) as adults, and maintained them in egg-laying condition by allowing them to mate every 8 days until death. Within each treatment, we allocated females into subgroups, that is, life-history females (to record life-history data) and RNA-1 and RNA-2 females (to characterize gene expression at 10% and 60% mortalities, respectively). We used these subgroups to test the effect of larval diet on individual longevity and fertility, and on age-related gene expression, in adult females (Figure 1; see also Supplementary Methods for further details).

### Aim 1: Effect of Larval Diet Treatments on Fecundity–Longevity Relationships

We used the 157 (56 L + 45 M + 56 H) life-history females to determine the effects of treatment on longevity and fertility for each female from day 4 (Figure 1). For longevity, we twice daily (between 09:30–11:00 am and 17:00–19:00 pm) recorded whether each female was dead (until the last female death 88 days eclosion). For fertility, we obtained egg counts and adult offspring counts (eggs that reached adulthood) every 24 hours for 7 consecutive days over the first 8-day cycle (ie, 8 days following the first mating opportunity) and every 48 hours over subsequent 8-day cycles (ie, 8-day periods following later mating opportunities). Once each female had died, we recorded thorax size as an index of body size (see Supplementary Methods for further details).

### Aim 2: Effect of Larval Diet Treatments on Gene Expression in Adult Females

We used the RNA-1 and RNA-2 subgroups of females to produce samples for gene expression profiling by mRNA-seq (Figure 1). Within each treatment and subgroup, we recorded mortality as for the life-history females and collected surviving females after the requisite number of females had died (10% for the RNA-1 subgroup, 60% for the RNA-2 subgroup). We chose these relative (rather than absolute) mortality thresholds to account for differences in aging between different treatments and facilitate comparisons with other studies (33,37). Overall, we collected the following numbers of females from each treatment: L: RNA-1: *n* = 49 (out of 54), RNA-2: *n* = 48 (out of 120); M: RNA-1: 45 (out of 50), RNA-2: 37 (out of 92); H: RNA-1: *n* = 49 (out of 54), RNA-2: *n* = 48 (out of 120).

#### Dissections and RNA extraction

We sequenced the RNA-1 and RNA-2 mRNA samples from the 2 treatments that showed the most dissimilar fecundity–longevity relationships from each other (ie, the M and H treatments; see Results section). For each female, we dissected the head, fat body, and ovaries, and then pooled each tissue into samples of 11–12 individuals each. We then extracted RNA from each tissue sample, and sent 3 RNA samples of each treatment, tissue, and subgroup to Edinburgh Genomics (Edinburgh, UK) for Illumina 100 base pair, paired-end sequencing on a NovaSeq6000 sequencer (3 samples of 11–12 pooled individuals each × 3 tissues × 2 sub-groups × 2 treatments = 36 RNA samples in total).

### Statistical Analysis

To address our first aim (effect of larval diet treatments on fecundity–longevity relationships), we implemented the following statistical approaches to analyze fertility and longevity data from females of the life-history subgroups. All analyses were conducted with the R (version 4.0.3) statistical programming platform in RStudio (v.2022.02.0; see Supplementary Methods for further details).

#### Longevity

**Figure 2.**
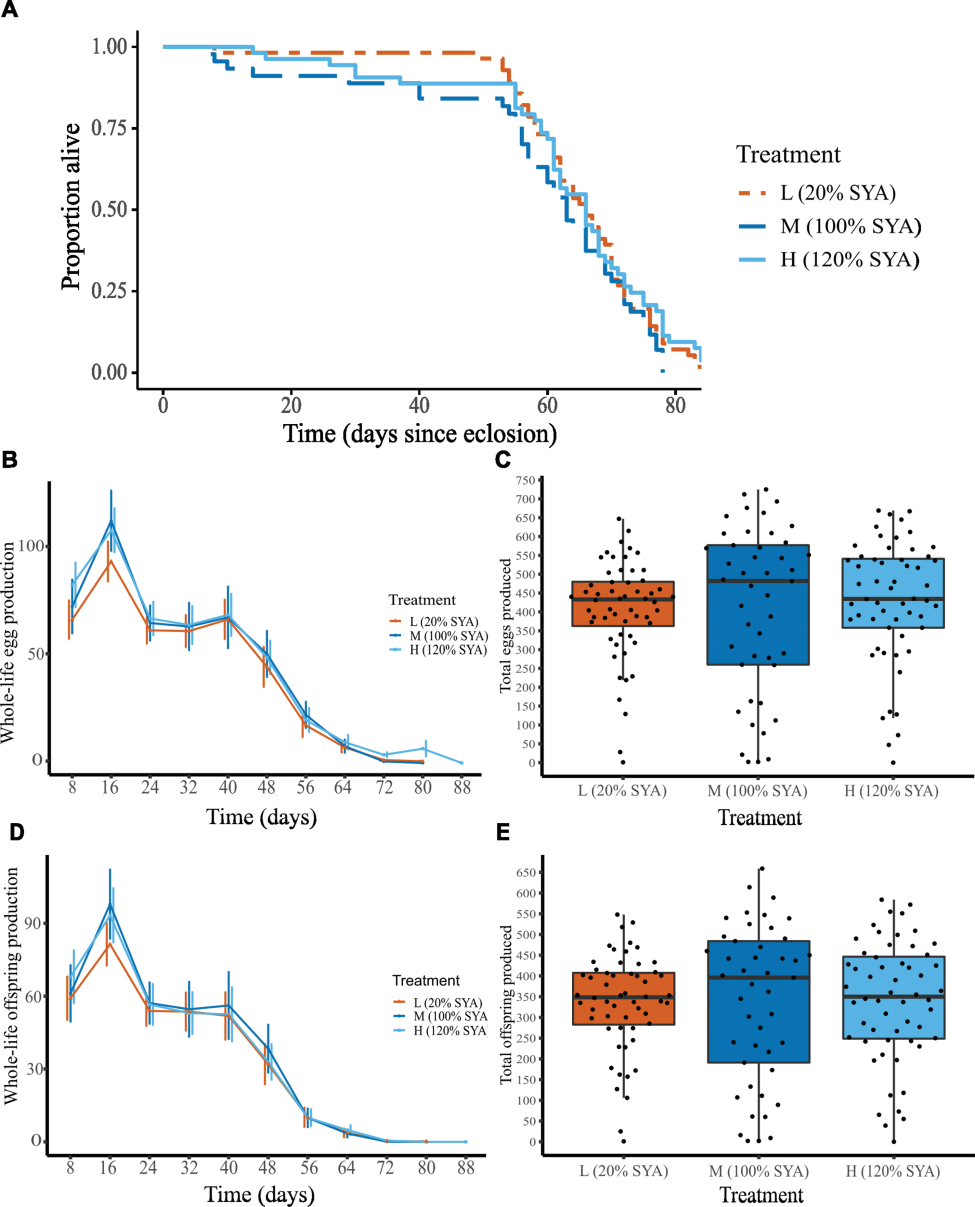
Longevity and fertility in adult female *Drosophila melanogaster* reared on L (20% Sugar Yeast Agar [SYA], orange, *n* = 56), M (100% SYA, dark blue, *n* = 45), H (120% SYA, light blue, *n* = 56) treatment diets as larvae, and on 110% SYA diet as adults. (A) Proportion alive as a function of time (days since eclosion) for L (orange dashed line), M (dark blue dashed line), and H (light blue solid line) females. There was no significant difference between treatments in proportion alive as a function of time. (B) Whole-life egg production (number of eggs produced per 8-day cycle) as a function of time (represented by the last day of each successive 8-day cycle) for L (triangles), M (circles), and H (squares) females. Error bars (offset): 1 *SD.* Whole-life egg production decreased significantly with time for all treatments, but there was no significant difference between treatments in whole-life egg production. Early-life egg production (ie, set of points at day 8) was significantly higher for H females compared to M and L females. (C) Total eggs produced (sum total number of eggs produced per female, black circles). Horizontal bars: median (across all females within each treatment); boxes: interquartile ranges; whiskers: ranges up to 1.5× the interquartile range. There was no significant difference between treatments in total eggs produced. (D) Whole-life offspring production (number of adult offspring produced per 8-day cycle) as a function of time (represented by the last day of each successive 8-day cycle) for L (triangles), M (circles), and H (squares) females. Error bars (offset): 1 *SD*. Whole-life offspring production decreased significantly with time for all treatments, but there was no significant difference between treatments in whole-life offspring production. There was also no significant difference between treatments in early-life offspring production (ie, set of points at day 8). (E) Total offspring produced (sum total number of eggs produced per female, black circles). Horizontal bars: median (across all females within each treatment); boxes: interquartile ranges; whiskers: ranges up to 1.5× the interquartile range. There was no significant difference between treatments in total offspring produced.

We investigated the effect of treatment on longevity (number of days between the date the female eclosed and the date of the female’s death) using Cox Proportional Hazards regression analyses. We first modeled longevity including all females and then, as differences in female survival appeared to converge after day 55 (Figure 2A), we conducted 2 additional analyses. These either included only females dying before day 55 (“early-death females”), with females dying after day 55 (“late-death females”) being censored, or included only late-death females, with early-death females being censored (see Supplementary Methods for further details). In each of these 3 analyses, treatment had no significant effect on longevity, so only the longevity model output from the analysis including all females is reported.

#### Fertility

We investigated the effect of treatment on fertility by analyzing 4 time-dependent (ie, repeated) measures of fertility for each life-history female: (1) early-life egg production: the number of eggs produced every 24 hours for the first 8-day cycle for each female; (2) early-life offspring production: the number of adult offspring produced every 24 hours for the first 8-day cycle for each female; (3) whole-life egg production: the number of eggs produced during each 8-day cycle (including the first 8-day cycle) across the whole life of each female; and (4) whole-life offspring production: the number of adult offspring produced during each 8-day cycle (including the first 8-day cycle) across the whole life of each female. We analyzed each of these measures using zero-inflated generalized linear mixed effect models with negative binomial error structure.

We also tested for an effect of treatment on 2 additional lifetime measures of fertility for each female: (1) total eggs produced: the total number of eggs produced (summed across all 8-day cycles) and (2) total offspring produced: the total number of adult offspring produced (summed across all 8-day cycles). We analyzed each of these measures using glms with negative binomial error structure.

Lastly, we analyzed the effect of treatment on each female’s egg viability (the total number of adults developing from eggs as a proportion of the total number of eggs that were produced during each 8-day cycle). We analyzed these data using a glmm with binomial error distribution.

For all fertility measures, the full list of fixed and random effects is described in Supplementary Methods and the model rankings (from lowest to highest AICc value) are reported in Supplementary Table 1.

#### Fecundity–longevity relationships

**Figure 3.**
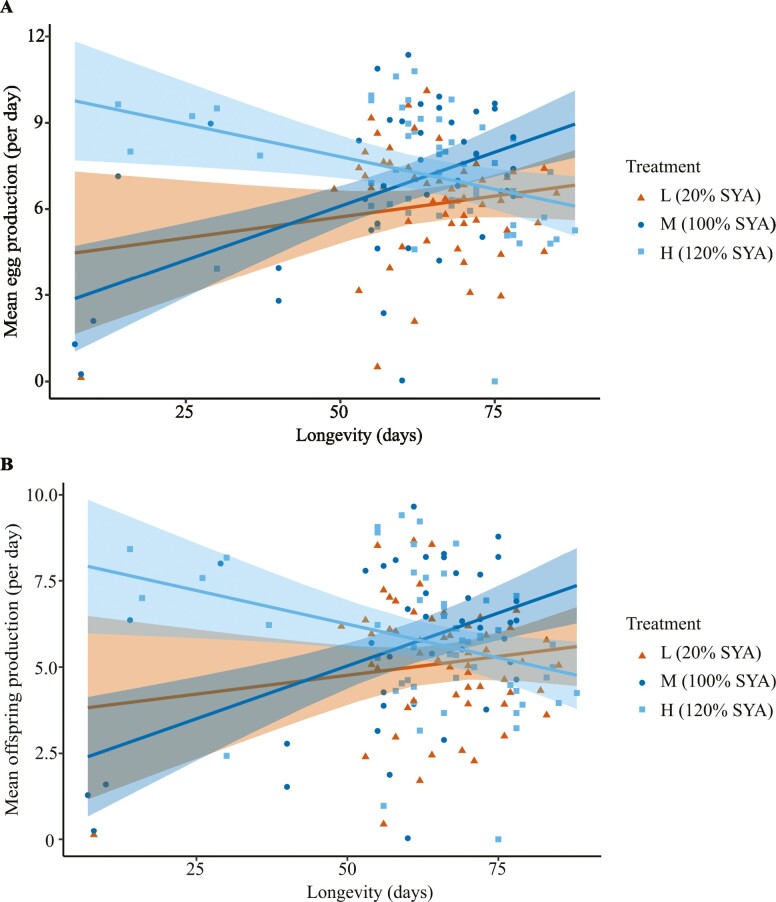
Fecundity–longevity relationships in adult female *Drosophila melanogaster* reared on L (20% Sugar Yeast Agar [SYA] diet, orange, *n* = 56), M (100% SYA diet, dark blue, *n* = 45), H (120% SYA diet, light blue, *n* = 56) treatment diets as larvae, and on 110% SYA diet as adults. (A) Longevity versus whole-life mean fertility measured from mean egg production (mean number of eggs produced per day over each female’s whole life). (B) Longevity versus whole-life mean fertility measured from mean offspring production (mean number of adult offspring produced per day over each female’s whole life). The solid line and shading represent each linear relationship and set of confidence intervals for each treatment, respectively. For both mean egg production and mean offspring production, females showed no significant fecundity–longevity relationship on 20% SYA diet, a significant positive relationship on 100% SYA diet, and a significant negative relationship on 120% SYA diet.

To test the hypothesis that differences in larval diet would change the directionality of the fecundity–longevity relationship within each treatment, we used ANCOVA to determine the effects of treatment and longevity on whole-life mean fertility (mean number of eggs or mean number of adult offspring produced per day over each female’s whole life). To check that the early-death females were not disproportionately contributing to the relationships found (Figure 3), we then repeated the analysis including only late-death females, with early-death females omitted. To check for any effects of fecundity early in life (given that in natural populations many females might die relatively young), we also repeated the analysis using early-life mean fertility (mean number of eggs or offspring produced per day over the first 8-day cycle) in place of whole-life mean fertility.

To explore potential effects of treatment further, we also investigated whether treatment affected development time, development success, and adult female thorax size (see Supplementary Methods for further details).

### Bioinformatic Analysis

To address our second aim (effect of larval diet treatments on gene expression in adult females), we analyzed the mRNA-seq data from the RNA-1 and RNA-2 subgroups of M and H females, having determined that each mRNA-seq library passed quality assessment (Supplementary Figures 1–3, Supplementary Table 2, and Supplementary Methods for further details).

#### Age-related gene expression

We estimated transcript counts for each gene and pseudoaligned reads to the *D. melanogaster* transcriptome (Supplementary Tables 3 and 4). We then conducted differential expression analysis using an FDR adjusted *p-*value threshold of .05, to generate 4 lists of differentially expressed genes (DEGs) for each tissue: genes more highly expressed in RNA-2 than RNA-1 (upregulated genes) and genes more highly expressed in RNA-1 than RNA-2 (downregulated genes) within each of the M and H treatments (Tables D1–D3 (43)).

We performed Fisher’s Exact Tests to detect significant overlaps between M and H treatment DEGs for each tissue (head, fat body, and ovaries) and each direction of differential expression with age (up- or downregulated genes), resulting in a total of 6 comparisons (see Supplementary Methods for further details).

#### Gene ontology

For each tissue, we identified biological processes Gene ontology (GO) terms that were significantly overrepresented (*p* < .05 following Benjamini–Hochberg correction for multiple testing) in a set of DEGs against a background consisting of all genes expressed in the tissue (see Supplementary Methods for further details).

#### Comparisons with other data sets

To further explore the effect of treatment on gene expression, we compared lists of M and H treatment DEGs from the current data set to lists of age- and/or aging-related genes from previous studies. We first compared lists of M and H treatment DEGs with (for head) DEG lists from Pacifico et al. (44) (brain) and (for fat body) those from Chen et al. (45) (fat body) (8 comparisons). We also compared lists of DEGs from the current data set to lists of *D. melanogaster* genes in the GenAge database (42) (6 comparisons) and the TI-J-LiFe network (27) (36 comparisons). For all these analyses, we used Fisher’s Exact Tests (with Bonferroni correction) to detect significant overlaps between gene lists (see Supplementary Methods for further details).

## Results

### Aim 1: Effect of Larval Diet Treatments on Fecundity–Longevity Relationships

#### Longevity

The longevities of L females (mean longevity (*SD*) = 65.2 (16.3) days), M females (mean longevity (*SD*) = 58.7 (19.1) days), and H females (mean longevity (*SD*) = 63.3 (11.7) days) did not differ significantly (coxph: M > L: *z* = −1.234, *p* = .217; M > H: *z* = −1.799, *p* = .072; Figure 2A). Therefore, the treatments had no effect on adult female survivorship and longevity.

#### Fertility

H females had significantly higher early-life egg production than M and L females (mean eggs produced per day (*SD*): H = 12 (7.9), M = 10.9 (8.5), L = 9.6 (7.3); glmm: *z* = 1.999, *p* = .046; Figure 2B; Supplementary Tables 1 and 5). However, there was no effect of treatment on whole-life egg production (glmm: H > M: *z* = 1.300, *p* = .194; M > L: *z* = 0.083, *p* = .934; Figure 2B; Supplementary Tables 1 and 5) or total eggs produced (LRT: *df* = 2, χ^2^ = 0.221, *p* = .895; Figure 2C). Despite the early-life differences in egg production, there were no significant differences between treatments in early-life offspring production (glmm: H > M: *z* = 1.227, *p* = .220; L > M: *z* = 0.206, *p* = .837), whole-life offspring production (glmm: H > M: *z* = 0.162, *p* = .871; L > M: *z* = 0.745, *p* = .474; Figure 2D; Supplementary Tables 1 and 5), or total offspring produced (LRT: *df* = 2, χ^2^ = 0.008, *p* = .996, Figure 2E). There were also no significant differences between treatments in egg viability (glmm: H > M: *z* = −0.578, *p* = .563; L > M: *z* = −0.030, *p* = .976; Supplementary Figure 4, Supplementary Tables 1 and 5). In all 3 treatments, whole-life egg production, whole-life offspring production, and egg viability decreased significantly over time (glmm: whole-life egg production: *z* = −35.994, *p* < .001; whole-life offspring production: *z* = −21.419 *p* < .001; egg viability: *z* = −14.067, *p* < .001; Figures 2B and D; Supplementary Figure 4, Supplementary Tables 1 and 5) and there were no interactions between treatment and time for any of these variables (Supplementary Tables 1 and 5).

#### Fecundity–longevity relationships

The relationship between longevity and whole-life mean fertility (mean egg or mean adult offspring produced per day over each female’s whole life) showed a significant interaction with treatment (ANCOVA, eggs: *F* = 11.991, *df* = 2, 146, *p* < .001; offspring: *F* = 9.427, *df* = 2, 146, *p* < .001; Figure 3). L females showed no significant relationship between longevity and whole-life mean fertility (eggs: *F* = 1.619, *df* = 1, 54, *p* = .209, *R*^2^ = 0.011; offspring: *F* = 1.096, *df* = 1, 54, *p* = .299, *R*^2^ = 0.002), M females a significant positive relationship (eggs: *F* = 14.63, *df* = 1, 41, *p* < .001, *R*^2^ = 0.245; offspring: *F* = 12, *df* = 1, 41, *p* = .001, *R*^2^ = 0.208), and H females a significant negative relationship (eggs: *F* = 7.664, *df* = 1, 51, *p* = .008, *R*^2^ = 0.114; offspring: *F* = 5.529, *df* = 1, 51, *p* = .023, *R*^2^ = 0.08; Figure 3). When only late-death females were included in the analysis, L females still showed no significant relationship between longevity and whole-life mean fertility, and H females still showed a significantly negative relationship, but M females showed no significant relationship (see Supplementary Results for further details). When early-life mean fertility (ie, mean number of eggs or mean number of offspring produced per day over the first 8-day cycle) was used in place of whole-life mean fertility, L females still showed no significant relationship between longevity and mean fertility and M females again showed a significant positive relationship, but H females showed no significant negative relationship (see Supplementary Results for further details). Overall, therefore, L, M, and H females exhibited fecundity–longevity relationships differing in directionality. Moreover, although the positive and negative relationships found, respectively, in M and H treatment groups, were nonsignificant in 1 of the 3 analyses, this overall finding remained the case for the analysis using whole-life mean fertility but including only late-death females and for the analysis using early-life mean fertility.

These results support the hypothesis that the directionality of the fecundity–longevity relationship in adult female *D. melanogaster* can be changed by larval diet. However, the signs of these relationships were not as we predicted for their respective diets (ie, more positive in H females than in M and L females), and therefore, the contrast in directionality was also not the same as the one hypothesized.

Treatment also significantly affected development time, with the rank order of development time (slowest first) being L, M, and H females (Supplementary Figures 5 and 6), and adult female thorax size, with L females having significantly smaller thorax sizes than M or H females; however, treatment did not significantly affect development success (Supplementary Figures 7–9; see Supplementary Results for further details).

### Aim 2: Effect of Larval Diet Treatments on Gene Expression in Adult Females

#### Age-related gene expression

**Figure 4.**
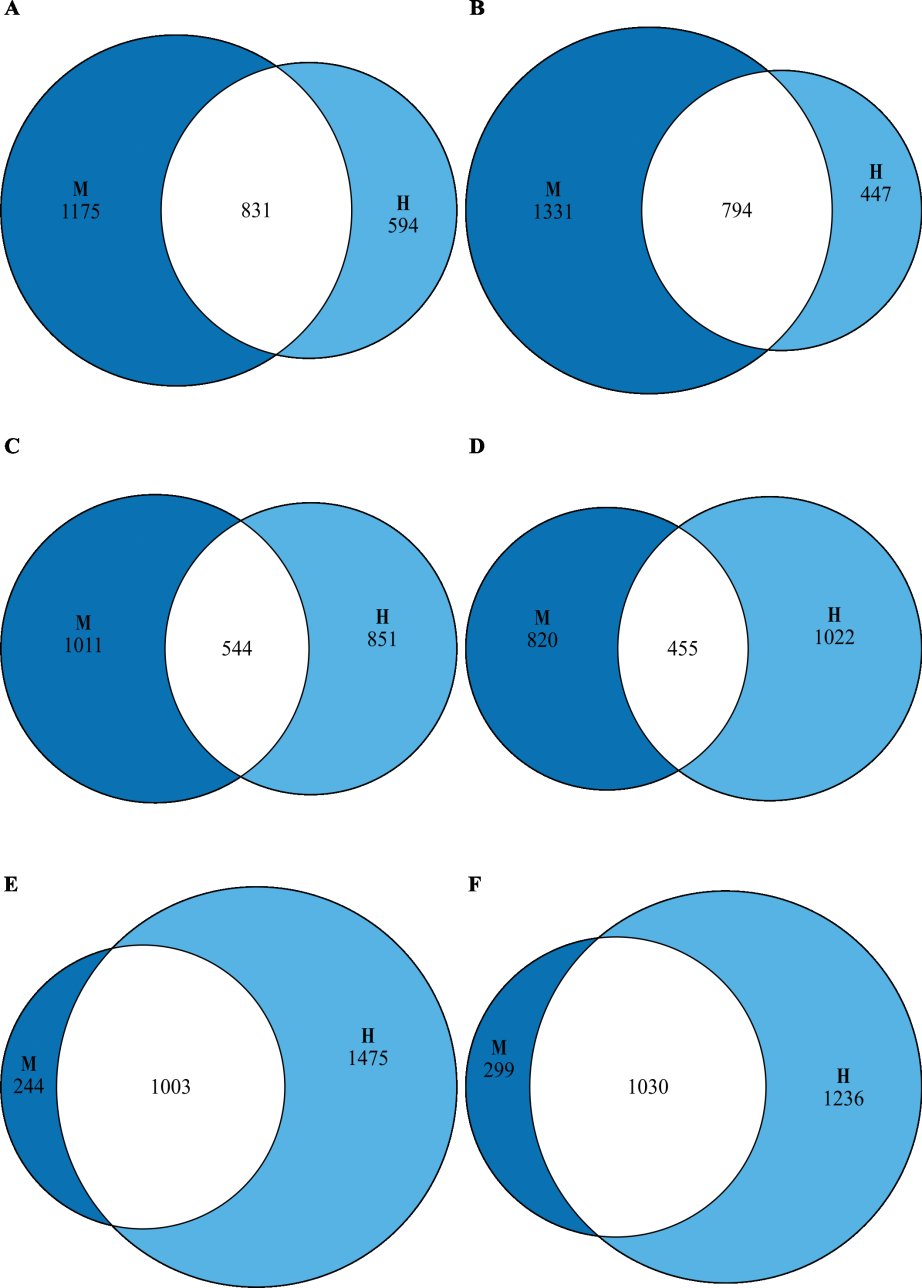
Comparison of changes in gene expression profiles with time in 3 tissues between M (100% Sugar Yeast Agar [SYA]) and H (120% SYA) adult female *Drosophila melanogaster* as determined by mRNA-seq. Euler diagrams of overlaps between differentially expressed genes (DEGs), ie, genes differentially expressed between RNA-1 females and RNA-2 females within treatments and shared between M (dark blue circles) and H (light blue circles) treatments for: (A, B), head; (C, D), fat body; and (E, F), ovaries. All 6 comparisons showed significant overlap in DEGs (Fisher’s Exact Tests, *p* < .05 after Bonferroni correction). Upregulated DEGs (left-hand diagrams): DEGs significantly more expressed in RNA-2 females than RNA-1 females, that is, that increase expression with age; downregulated DEGs (right-hand diagrams): DEGs significantly more expressed in RNA-1 females than RNA-2 females, that is, that decrease expression with age. Results of statistical tests are in Supplementary Table S6 and identities of overlapping genes are in Table D4 (43).

In head, 41.4% of upregulated M DEGs were shared with upregulated H DEGs, and 37.4% of downregulated M DEGs were shared with downregulated H DEGs. In fat body, 35.0% of upregulated M DEGs were shared with upregulated H DEGs, and 35.7% of downregulated M DEGs were shared with downregulated H DEGs. In ovaries, 80.4% of upregulated M DEGs were shared with upregulated H DEGs, and 77.5% of downregulated M DEGs were shared with downregulated H DEGs. Overall, all comparisons (6/6) of M and H DEGs showed significant overlaps (mean percentage overlap relative to the M treatment = 51.23%; Fisher’s Exact Test, *p* *<* 1 × 10^−124^ in all cases; Figure 4; Supplementary Figures 10–12, Supplementary Table 6; Table D4 (43)).

Hence, there were significant similarities between treatments in age-related gene expression in all 3 tissues, but, in head and fat body, most DEGs were nonoverlapping between treatments.

#### Gene ontology

Gene ontology enrichment analysis isolated 1 323 nonredundant enriched GO terms for the DEGs across all treatments and tissues (Supplementary Table 7; Table D5 (43)). For the aging-specific GO terms GO:0007568 “aging” and/or GO:0010259 “multicellular organism aging,” we found enrichment in DEGs upregulated in the head of M and H females, and in DEGs downregulated in the fat body and ovaries of H females (Supplementary Table 7). For other GO terms, GO enrichment analysis showed substantial differences in GO terms associated with genes upregulated and downregulated with age in M and H females (Supplementary Table 7; see Supplementary Results for further details). Hence, the biological functions of age-related DEGS (including those related specifically to aging) differed between each treatment.

#### Comparisons with other data sets

In the comparisons with previously isolated *D. melanogaster* age-related DEGs from Pacifico et al. (44) (brain) and Chen et al. (45) (fat body), 7/8 comparisons showed significant overlap between M and H treatment DEGs and DEGs in the corresponding previous data set (mean [range] percentage overlap in significant comparisons = 16.14% [10.2%–21.2%] of DEGs in the current data set; Fisher’s Exact Test, *p* < 10^−6^ in each case; Supplementary Table 8; Table D6 (43)). Only the comparison between DEGs upregulated in fat body in H females and in the Chen et al. (45) data set did not show significant overlap (Fisher’s Exact Test, *p* = .03; Supplementary Figure 13, Supplementary Table 8; Table D6 (43)). However, within the current data set, the treatments differed in level of overlap with the previous data sets, with the H treatment showing, relative to the M treatment, greater overlap in head (M upregulated = 10.9%, M downregulated = 10.2%; H upregulated = 16%, H downregulated = 19.4%) and less overlap in fat body (M upregulated = 21.2%, M downregulated = 12.7%; H upregulated = no significant overlap, H downregulated = 11.4%).

For the comparisons with genes in the *D. melanogaster* GenAge database (42), 2/6 comparisons showed significant overlap. The 2 significant overlaps were both in M females, in which 98/194 (50.5%) of the GenAge genes were upregulated in head (Fisher’s Exact Test, *p* = 1.01 × 10^−7^) and 68/194 (35.1%) of the GenAge genes were upregulated in fat body (Fisher’s Exact Test, *p* = 9.46 × 10^−5^; Supplementary Figure 13, Supplementary Table 9; Table D7 (43)).

**Figure 5.**
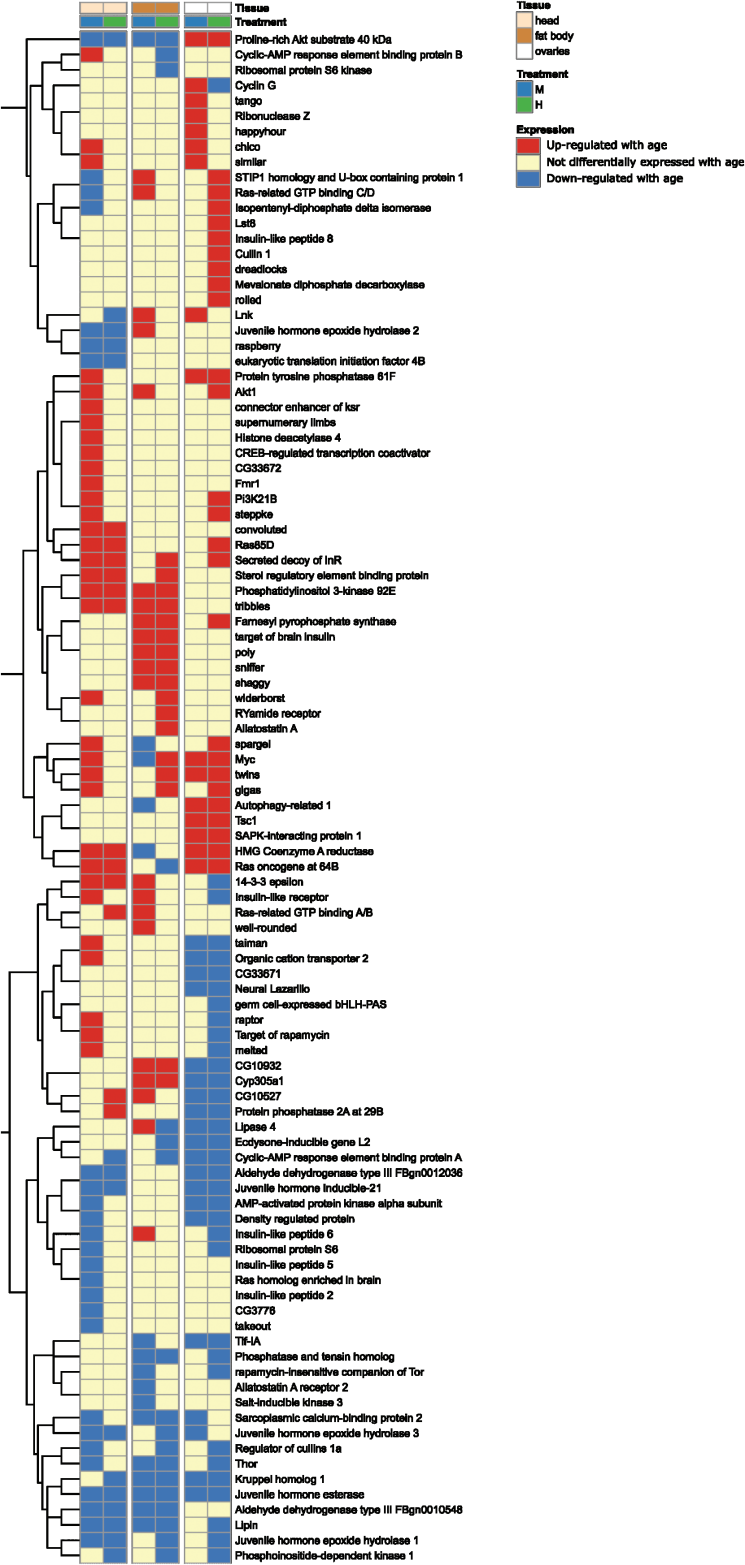
Results of comparison of age-related genes in M (100% SYA) and H (120% SYA) adult female *Drosophila melanogaster* and genes in the TI-J-LiFe network. Each row represents an individual gene from *D. melanogaster* described as a component of the TI-J-LiFe network by Korb et al. (27). Each column shows the age-related expression status of focal genes in a given treatment and tissue in *D. melanogaster* females in the current study. Vertical breaks separate the 3 tissues studied (head, fat body, and ovaries). The dendrogram at left groups genes according to their gene expression patterns.

For the comparisons with genes in the TI-J-LiFe network (27), we found that, in head in M females, for 1/6 comparisons, 59/123 (48%) of the TI-J-LiFe genes significantly overlapped with DEGs in the current data set (Fisher’s Exact Test, *p* = .00003). The significant comparison occurred when all M-head DEGs were included in the analysis (Supplementary Table 10). However, there were no significant overlaps with TI-J-LiFe genes in any other tissue or treatment combinations (0/30 comparisons showed overlap; Figure 5; Supplementary Table 10; Table D8 (43)).

Overall, DEGs in the M and H treatments were similar but not identical in comparisons with age-related genes from 2 previous studies of *D. melanogaster*. Moreover, only M treatment DEGs showed significant overlaps in gene expression with the GenAge and TI-J-LiFe lists of aging-related genes.

## Discussion

We tested for an effect of larval diet on the directionality of the fecundity–longevity relationship (aim 1) and for associated age-related gene expression changes (aim 2) in adult *D. melanogaster* females. We found that larval diet significantly affected development time and adult female thorax size, but not development success (Supplementary Figures 5–9). Larval diet had no effect on adult female survivorship and longevity (Figure 2A) and only relatively small effects on fecundity (no difference in whole-life egg production and modest increase in H females in early-life egg production; Figure 2B). Despite this, we found for the first time that changes in larval diet altered the directionality of the fecundity–longevity relationship in adults (Figure 3). All 3 analyses conducted showed a significant interaction of the fecundity–longevity relationship with larval diet treatment. Specifically, L females showed no relationship between fertility and longevity, M females showed a significant positive relationship (or no relationship in the analysis including only late-death females) and H females showed a significant negative relationship (or no relationship when early-life mean fertility rather than whole-life mean fertility was analyzed). Larval diet also had a marked effect on age-related gene expression, with M and H females exhibiting significantly overlapping, but distinct, sets of genes differentially expressed with age, as well as different associated GO terms (Figures 4 and 5; Supplementary Figure 13, Supplementary Tables 7–10; Tables D6–D8 (43)).

### Effect of Larval Diet Treatments on Fecundity–Longevity Relationships

Other things equal, the standard evolutionary theory of aging predicts a negative fecundity–longevity relationship; however, there are several classes of exceptions (see Introduction section). *Drosophila melanogaster* typically exhibits a negative fecundity–longevity relationship (4), although fecundity and longevity can become uncoupled in long-lived mutants (8,11,46) and on different quality adult diets (30,35). Our experiment shows that the fecundity–longevity relationship can also change directionality depending on larval diet quality. However, such changes did not result in longer-lived females incurring greater costs of reproduction, as, although H females had greater early-life egg production, longevity across all treatments did not differ significantly. Previously, diet and body size have been shown to interact in their effects on longevity (34); however, we found that adult female thorax size was not a significant predictor of longevity, fecundity, or fecundity–longevity relationships (see Supplementary Methods for further details). An earlier study also showed that, as in the current one, varying the yeast content of larval diet did not affect adult mortality (40). In addition, this earlier study found that adult females reared on a low-yeast larval diet showed lower fecundity because of a decrease in their ovariole number (40). Differences in ovariole number (though not investigated) may have been present in the current study, but it seems unlikely that such differences strongly affected fecundity as the only significant effect of treatment on fecundity was that H females had greater early-life egg production (Figure 2B). Nonetheless, our study matches the earlier study (40) in finding that fecundity differences between treatments were not associated with differences in adult mortality. In the current study, all females also showed reproductive senescence, that is, their fertility declined significantly with time in all treatments (Figure 2B).

Our results could help explain why the fecundity–longevity relationship is positive in other systems. Specifically, in eusocial insects, positive fecundity–longevity relationships could be explained by high-quality individuals, which received superior larval provisioning, being able to reproduce at higher rates without expressing longevity costs of reproduction (18). Previous studies in *D. melanogaster* have shown that increased larval diet quality can increase female fertility and longevity (32,33,39), and the current study has now shown that larval diet quality can affect the directionality of the fecundity–longevity relationship. Therefore, asymmetric resourcing via changes in larval diet quality could help explain why eusocial insect reproductives show positive fecundity–longevity relationships.

Although our results showed that larval diet quality affected the directionality of the fecundity–longevity relationship, the specific patterns observed differed from those predicted, that is, that fecundity–longevity relationships would be negative in L and M females and less negative or positive in H females. One explanation is that females reared on high-quality diet had greater early reproductive investment and lower mortality rate (as H females had significantly higher early-life egg production than M females (Figure 2B) and lower (albeit nonsignificantly) early mortality. Therefore, H females performing poorly (in terms of longevity) might still have been able to live beyond the first few weeks because of their higher quality diet. However, this explanation assumes that low-quality individuals can increase their reproduction without costs on high-quality diets, which would require testing.

A second explanation is that mismatches between larval and adult diets are costly. For example, *D. melanogaster* females transferred from low-quality (20% SYA) larval diet to high-quality (120% SYA) adult diet had greater fecundity and longevity compared to females transferred from high-quality larval diet to low-quality adult diet (39). However, this explanation seems unlikely to account for the current results because: (1) larva-to-adult dietary mismatches for the treatments showing the largest difference in directionality of their fecundity–longevity relationships (H and M) were relatively small (H: 120% to 110% SYA; M: 100% to 110% SYA); and (2) the treatment with the largest dietary mismatch (L: 20% to 110% SYA) did not generate a fecundity–longevity relationship opposite to those of the other treatments.

A third explanation is that females experienced the M diet (100% SYA) as the highest quality diet. In support of this, the M diet is the standard *D. melanaster* diet (47,48); the populations used for this study had been reared on it for 20 years; and *D. melanogaster* is known to show evolved responses to rearing diet (49). Furthermore, this explanation could also underlie the nonlinear effect of diet quality on the fecundity–longevity relationship in the current study (ie, L: no relationship; M: significantly positive relationship or no relationship; H: significantly negative relationship or no relationship). Similarly, previous studies of *D. melanogaster* have reported complex, nonlinear effects of adult and larval diet on fecundity and longevity (33,48,50).

### Effect of Larval Diet Treatments on Gene Expression in Adult Females

As well as showing dissimilar fecundity–longevity relationships, M and H treatments differed in their age-related gene expression profiles. Specifically, they exhibited: (1) despite significant overlap, substantial differences in age-related gene expression in head and fat body (Figure 4); (2) dissimilar GO terms with respect to biological functions of age- and aging-related genes (Supplementary Table S7), with, for example, in fat body and ovaries, downregulated DEGs being enriched for the GO term “multicellular organism aging” in H but not M females (Supplementary Table ); and (3) contrasts in the level of overlap between their age-related genes and age-related genes from 2 previous studies of *D. melanogaster* (44,45) and aging-related genes from the GenAge database (42) and TI-J-LiFe network (27) (Figure 5; Supplementary Figure 13).

For genes in the GenAge database, the largest (and only significant) overlaps with our data were for upregulated genes in M females in head and fat body. For genes in the TI-J-LiFe network, only one of the comparisons (also in M females) showed significant overlap, with this comparison showing high overlap (48%) with the TI-J-LiFe gene list. Overall, the similarities between age-related genes in the current data set and age- and/or aging-related genes in the comparison data sets demonstrate congruence across different studies in the genes and gene pathways returned as underpinning aging in *D. melanogaster*. However, the differences between M and H females in the current data set in comparisons with the other data sets also show that larval diet can strongly affect age-related gene expression in adult females and, in particular, demonstrate an association between altered fecundity–longevity relationships and differences in age-related gene expression. It remains to be tested whether the observed changes in the directionality of the fecundity–longevity relationship caused the observed changes in age-related gene expression or vice versa.

In conclusion, this study shows that experimental manipulation of larval diet can change the directionality of the fecundity–longevity relationship in adult female *D. melanogaster* and that such changes are accompanied by changes in expression of age- and aging-related genes. These results were observed even under relatively modest differences in larval diets and even when mean fertility and longevity showed minimal differences between treatments, although, as the results are from a single experiment, establishing the robustness of such effects would require replication. As well as demonstrating the plasticity of fecundity–longevity relationships, these findings suggest possible mechanistic causes of otherwise puzzling positive fecundity–longevity relationships in other systems, notably eusocial insects. Hence, a key outstanding question is whether larval diet quality affects the nature of the fecundity–longevity relationships found in these other systems.

## Supplementary Material

glad199_suppl_Supplementary_Material

## Data Availability

Data on differentially expressed genes, redundant GO terms, and overlapping genes from gene expression comparisons (Tables D1–D8) are available in Collins et al. (43). Data on longevity, fertility, morphometrics, etc., from this study are available in Collins et al. (51). The raw mRNA-seq sequencing data from this study have been deposited in the National Center for Biotechnology Information’s (NCBI’s) Gene Expression Omnibus (GEO) at https://www.ncbi.nlm.nih.gov/geo/ and are available under series accession number GSE175623. The code used for the analyses is available at https://zenodo.org/record/8211092 (DOI: 10.5281/zenodo.8211092).
